# Patient experiences of outcomes of bariatric surgery: a systematic review and qualitative synthesis

**DOI:** 10.1111/obr.12518

**Published:** 2017-03-08

**Authors:** Karen D. Coulman, Fiona MacKichan, Jane M. Blazeby, Amanda Owen‐Smith

**Affiliations:** ^1^ School of Social and Community Medicine University of Bristol Bristol UK; ^2^ Division of Surgery, Head and Neck University Hospitals Bristol NHS Foundation Trust Bristol UK

**Keywords:** bariatric surgery, patient experience, qualitative, synthesis

## Abstract

Although bariatric surgery is the most effective treatment for severe and complex obesity, less is known about its psychosocial impact. This systematic review synthesizes qualitative studies investigating the patient perspective of living with the outcomes of surgery. A total of 2,604 records were screened, and 33 studies were included. Data extraction and thematic synthesis yielded three overarching themes: control, normality and ambivalence. These were evident across eight organizing sub‐themes describing areas of life impacted by surgery: weight, activities of daily living, physical health, psychological health, social relations, sexual life, body image and eating behaviour and relationship with food. Throughout all these areas, patients were striving for control and normality. Many of the changes experienced were positive and led to feeling more in control and ‘normal’. Negative changes were also experienced, as well as changes that were neither positive nor negative but were nonetheless challenging and required adaptation. Thus, participants continued to strive for control and normality in some aspects of their lives for a considerable time, contributing to a sense of ambivalence in accounts of life after surgery. These findings demonstrate the importance of long‐term support, particularly psychological and dietary, to help people negotiate these challenges and maintain positive changes achieved after bariatric surgery.

## Introduction

The World Health Organization reported that in 2014 over 600 million people worldwide, or roughly 13% of adults, were obese (body mass index [BMI] of ≥30). This represents a doubling of figures since 1980 1. The health risks of obesity have been well documented, including an increased risk of type 2 diabetes, cardiovascular disease, certain types of cancer, depression, reduced health‐related quality of life (HRQL) and premature death 2, 3, 4, 5, 6, 7, 8, 9, 10. Systematic reviews of quantitative evidence have shown that obesity (bariatric) surgery is the most effective treatment for severe and complex obesity, defined as a BMI ≥40, or between 35 and 40 with another significant disease that could be improved by weight loss, such as diabetes 11, 12, 13, leading to greater weight loss and improvement in some obesity‐related comorbidities (such as diabetes) in the short‐term (up to 2 years post‐surgery), compared with other interventions (lifestyle or drug therapy). Far less data are available with regard to the long‐term outcomes 12, 14, although there is evidence that some patients experience weight re‐gain, which can negatively impact physical and psychological health and HRQL 15, 16, 17, 18, 19, 20. While previous quantitative research mainly focuses on the clinical outcomes of bariatric surgery, previous qualitative research with bariatric surgery patients has provided detailed accounts of the psychosocial impacts of the surgery 21, 22, 23, 24.

Qualitative research can provide valuable insight into patients' experiences of living with the outcomes of a health treatment, in particular the complexity and depth of the lived experience 25. In particular, the qualitative literature highlights the variability and complexity of psychosocial changes associated with surgery and weight change (both gain and loss) 20, 26. However, the current published qualitative literature tends to report on small, single‐centre samples, with individual studies focusing on one or two specific areas impacted by bariatric surgery, such as body image or relationship with food (e.g.^27^, ^28^), rather than the full spectrum of outcomes experienced.

It is increasingly being recognized that there should be some attempt to synthesize the understandings gained from these isolated studies to inform the evidence base, as is commonly performed for quantitative research 29, 30, 31. Qualitative synthesis offers a way of bringing together disparate studies and overcoming issues of sample size and focus, generating clinically useful knowledge. Qualitative synthesis has been defined as ‘the bringing together of findings on a chosen theme, the results of which should, in conceptual terms, be greater than the sum of parts’ 29. The aim is not solely to aggregate findings as in quantitative meta‐analyses but to generate new insights that can be used to influence policy and practice, and generate new research questions 29, 32, 33, 34. Qualitative research studies have not been included in previous systematic reviews of bariatric surgery and are often not included in systematic reviews of quantitative evidence more generally, because of the difficulty in synthesizing the findings with quantitative evidence 12, 14, 35.

There are now a number of published qualitative studies that have examined patients' perspectives of living with bariatric surgery, which when synthesized could provide useful knowledge to inform the evidence base and clinical practice. In this study, a systematic review of qualitative research was undertaken to synthesize what is currently known about the patient perspective of living with the outcomes of bariatric surgery. This was undertaken as part of a larger study that aimed to develop a core outcome set for bariatric surgery 36 and to generate new insights on the outcomes of bariatric surgery, which could be used to influence clinical practice and future research.

## Methods

A synthesis of relevant qualitative studies was undertaken. The study had three main steps (i) systematic identification of studies; (ii) study appraisal and data extraction; and (iii) inductive thematic synthesis of study findings.

### Systematic identification of relevant studies – search strategy and selection criteria

The first author (K. C.) conducted a series of electronic searches in May 2014 in the Ovid versions of MEDLINE, EMBASE, PsycINFO, the Cochrane Library, CINAHL and Web of Science (including Science Citation Index Expanded, Social Sciences Citation Index and Arts & Humanities Citation Index). The search strategies combined search terms for bariatric surgery, with terms for qualitative research **(** **Supporting Information** **)**. There were no limits for study design or language. Search results were downloaded and managed within Endnote software 37. K. C. screened all abstracts, and full‐text articles were obtained for those that were potentially relevant. Exclusion criteria included (i) participants had not yet undergone bariatric surgery; (ii) experiences of surgery‐specific issues were not investigated; (iii) qualitative methods were not used. Review articles, conference abstracts and theses with no full‐text article published were excluded. Non‐English language articles were translated.

All included articles were double‐checked by the fourth author (A. O. S.) to ensure they met the inclusion criteria. To identify additional relevant studies, the reference lists of included studies were examined, and the journal *Qualitative Health Research* was hand searched. Additionally, relevant experts in the field (Dr Lindsey Bocchieri‐Ricciardi, Prof Jane Odgen and Dr Karen Throsby) were contacted to identify any additional studies not found through the other search methods.

### Appraisal and data extraction

Study appraisal and data extraction were carried out concurrently using a modified version of the Critical Appraisal Skills Program criteria for quality appraisal of qualitative research, which was modified for use in this study (available upon request from the authors) 29. Currently, there is considerable debate as to whether quality appraisal of qualitative research should be undertaken in order to exclude certain studies from reviews 38, 39. Some researchers have found that excluding poor quality studies from qualitative systematic reviews had no meaningful impact on their synthesis findings, as these studies contributed relatively little to the synthesis 30, 38. In this review, quality appraisal was used to facilitate thorough understanding of the studies and was not used to discard any studies. Initially, appraisal and data extraction were carried out independently by K. C. and A. O. S. on five of the studies. Their results were compared and discussed in order to resolve any differences in interpretation of the questions on the data extraction form. Minor changes were then made to the data extraction form. K. C. then carried out appraisal and data extraction on the remainder of the studies, and any queries that arose were discussed with A. O. S.

### Inductive thematic synthesis

**Figure 1:**
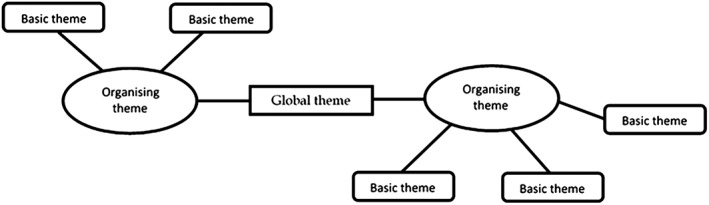
Example of a thematic network.

An inductive thematic synthesis was undertaken, broadly on the basis of the thematic analysis for synthesizing qualitative studies described by Thomas and Harden 30. This includes a process of translating concepts or themes from one study to another, similar to the reciprocal translation technique used in meta‐ethnography, first described by Noblit and Hare and applied to health research by Campbell *et al.* and Malpass *et al.* 29, 40, 41. A process of thematic networking was used to map and link themes into basic, organizing and global themes (Fig. 1) 34, 42. Themes reported by the authors of each study were extracted and listed (using authors' original wording) as a separate row in a spreadsheet. Findings from individual studies were then used to populate the columns of the spreadsheet, and a process of reciprocal translation was undertaken, whereby each study was scrutinized for evidence of all themes arising. Throughout this process, the description and wording of the themes were continually revised, and notes made as to how themes related and how some could be merged. Initial thematic networks were drawn to facilitate understanding of the themes, and broad organizing themes were identified 42. Each organizing theme was written up descriptively, and three global themes were identified.

## Results

### Study characteristics

**Figure 2:**
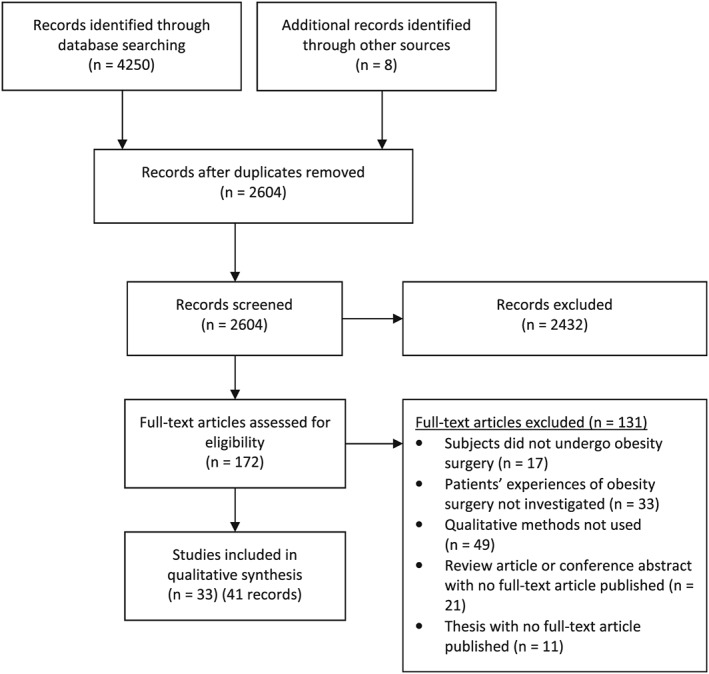
PRISMA systematic review diagram for qualitative synthesis. PRISMA = Preferred Reporting Items for Systematic Reviews and Meta‐Analyses.

Of 2,604 records screened, 41 papers relating to 33 studies met the inclusion criteria to be included in the review (Fig. 2). Detailed characteristics of included studies are presented in Table 1. Included studies were published between 2002 and 2014. Twelve studies (36.4%) were from the USA and Canada, eight (24.2%) from Scandinavia, six (18.2%) from Brazil, five (15.2%) from the UK, one (3.0%) from the Netherlands and one (3.0%) from New Zealand. Four studies were translated from Portuguese.

**Table 1:** Characteristics of included studies in qualitative synthesis

Study	Focus of investigation	Setting	Sample size and gender	Type of surgery and time since surgery	Data collection method
Bocchieri *et al.*, 2002 21; Meana and Ricciardi, 2008 22	Psychosocial experiences following gastric bypass	Hospital	33 (24 women)	RYGB, 6 months–10 years	Interviews (*n* = 22), focus groups (*n* = 11)
Ogden *et al.*, 2005, 2006 23, 43	Post‐surgery HRQL and eating behaviour	Hospital	15 (14 women)	Variety: gastric banding, gastric bypass and vertical gastroplasty, 4–33 months	Interviews
Wysoker, 2005 44	Individual experiences of bariatric surgery	Not reported	8 (5 women)	Type not reported, ≥1 year (unclear length of time),	Interviews
Earvolino‐Ramirez, 2008^45^	Case study of gastric bypass surgery	Not reported	1 woman	Gastric bypass, 8 months	Case study – interview
Pastoriza and Guimarães, 2008^46^	Behavioural change following bariatric surgery	Not reported	8 (7 women)	Capella method restrictive malabsorptive surgery, 1–5 years	Interviews
Throsby, 2008, 2009 47, 48, 49, 50	Discourse of re‐birth in the context of bariatric surgery	Community	35 (29 women)	Not reported	One focus group, then interviews
Agra and Henriques, 2009 51	Post‐surgery HRQL	Private gastroenterology practice	16 women	Gastroplasty, ‘medium‐term post‐op period’ (time not specified)	Interviews
Norris, 2009 52	Outcomes of bariatric surgery	Hospital	1 woman	Gastroplasty or bypass – unclear, interviewed at 2,6,12 and 18 months post‐op	Case study – longitudinal interviews
Sutton *et al.*, 2009 53	Individual experiences of bariatric surgery	Not reported	14 women	RYGB, >12 months	Interviews
Zijlstra *et al.*, 2009 19	Outcomes of bariatric surgery	Hospital	11 (10 women)	AGB, 2–5 years	Interviews
Groven *et al.*, 2010, 2012 26, 54	Side effects of bariatric surgery and bodily change	Health clinic and community	22 women	Gastric bypass, 5–6 years	Interviews‐two participants interviewed 1 year later
LePage, 2010 55	Individual experiences of bariatric surgery	Bariatric healthcare practices	12 (8 women)	RYGB, 2–9 years	Interviews
Magdaleno *et al.*, 2010, 2011 56, 57, 58	Discourse of transformation in the context of bariatric surgery	Hospital	7 women	Type not reported, 18 months–3 years	Interviews
Wilson, 2010 59	Outcomes of bariatric surgery	Personal reflection of author who underwent surgery	1 woman (author)	Type not reported, 12 months	Kept notes of her own experiences
Engström and Forsberg, 2011 60	Expectations and outcomes of bariatric surgery	Hospital	16 (12 women)	RYGB and BPD‐DS, interviewed pre‐op, 1, 2 years post‐op	Longitudinal interviews
Marcelino and Patrício, 2011 61	Outcomes of bariatric surgery	Not reported	6 (5 women)	Gastroplasty, time not reported	Interviews
Ogden *et al.*, 2011 20	Lack of success and revision procedures	Obesity clinic and a patient support group	10 (8 women)	Variety: band then bypass (*n* = 4), band then sleeve (*n* = 2), band awaiting bypass (*n* = 1), bypass followed by pouch revision (*n* = 2), bypass only (*n* = 1), 1–10 years since initial operation	Interviews
Throsby, 2012 62	Bodily discourses in the context of bariatric surgery	Hospital	153 patient consultations observed (103 women), plus 8 seminars, 15 interviews (11 women)	Gastric banding, except 3 gastric bypass, time not reported	Observations of clinics and seminars, interviews
Ivezaj *et al.*, 2012 63	Substance abuse and bariatric surgery	Substance abuse treatment programme	24 (18 women)	RYGB, mean time since surgery 5.5 (± 3.1 years)	Interviews
Zunker *et al.*, 2012 64	Eating behaviours post‐surgery	Community and via a research institute	29 (27 women)	Mostly RYGB, others not specified, 1–14 years, mean 8 years, median 2 years	Structured focus groups – nominal group technique
Benson‐Davies *et al.*, 2013 28	Outcomes of bariatric surgery	Community	18 women	RYGB, mean 75.0 ± 32.4 months (6.25 years)	Focus groups
Castro *et al.*, 2013 65	Body image following bariatric surgery	Diabetes and hypertension service	20 women	Gastroplasty, mean 2.85 years (± 0.988)	Interviews
Gilmartin, 2013 27	Body image following bariatric surgery	Hospital	20 (18 women)	Type not reported, 2–5 years	Interviews
Gronning *et al.*, 2013 66	Decision‐making around bariatric surgery	Hospital	12 (10 women)	RYGB (*n* = 10), AGB (*n* = 1), both RYGB and AGB (*n* = 1), time not reported	Interviews
Knutsen *et al.*, 2013 67	Empowerment discourses in the context of bariatric surgery	Hospital	9 (8 women)	RYGB, interviewed twice pre‐op, and at 2 weeks, 2–3 months, 9 months post‐op	Longitudinal interviews
Mariano *et al.*, 2013 68	Outcomes of bariatric surgery	Hospital	30 (24 women)	RYGB, mean 5.7 years (± 1.3)	Interviews
Natvik *et al.*, 2013 69	Outcomes of bariatric surgery	Hospital	8 (4 women)	Duodenal switch, 5–7 years	Interviews
Stolzenberger *et al.*, 2013 24	Post‐surgery HRQL	Hospital	61 (48 women)	RYGB (72%), AGB, 2–9 years	Focus groups
Forsberg *et al.* 2014 70	Expectations and outcomes of bariatric surgery	Hospital	10 (8 women)	RYGB, 1–2 months	Interviews
Geraci *et al.*, 2014 71	Outcomes of bariatric surgery	Community	9 women	SG (*n* = 7) and RYGB (*n* = 2), 2.5–7.5 years	Interviews
Jensen *et al.*, 2014 72	Body image following bariatric surgery	Hospital and community	5 women	RYGB, 1–12 months	Interviews
Lyons et al., 2014 73	Body image following bariatric surgery	Hospital	15 (12 women)	Type not reported, mean 26.1 months	Focus groups
Warholm *et al.*, 2014 74	Outcomes of bariatric surgery	Hospital	2 women	BPD‐DS, interviewed at 3, 6, 9 and 12 months post‐op	Longitudinal interviews

AGB, adjustable gastric band; BPD‐DS, biliopancreatic diversion with duodenal switch; HRQL, health‐related quality of life; RYGB, Roux‐en‐Y gastric bypass; SG, sleeve gastrectomy.

The majority of studies used one‐off individual interviews to collect data (*n* = 25, 75.8%), only five undertook longitudinal (repeated) interviews over periods of up to 2 years. Four studies (12.1%) used focus group discussion, and two (6.1%) used both interviews and focus groups. One study involved observation of clinic consultations and observation of seminars in addition to conducting interviews 62, and one documented her own experience in a personal notebook 59. Sample sizes ranged from 1 (three studies – one personal reflection and two case studies) to 61 for interview and focus group studies. Across all 33 studies, there were 656 participants recruited, the majority of which were women (529, 80.6%). Twenty‐three of the studies reported both the type of operation that participants underwent and the number of participants who underwent each type of operation. Among these, the majority of participants were reported to have undergone a Roux‐en‐Y gastric bypass (*n* = 248), followed by the adjustable gastric band (*n* = 45), gastroplasty (*n* = 43), duodenal switch (*n* = 10), sleeve gastrectomy (*n* = 9) and ‘capella method restrictive malabsorptive surgery’ (*n* = 8).

### Inductive thematic synthesis

**Figure 3:**
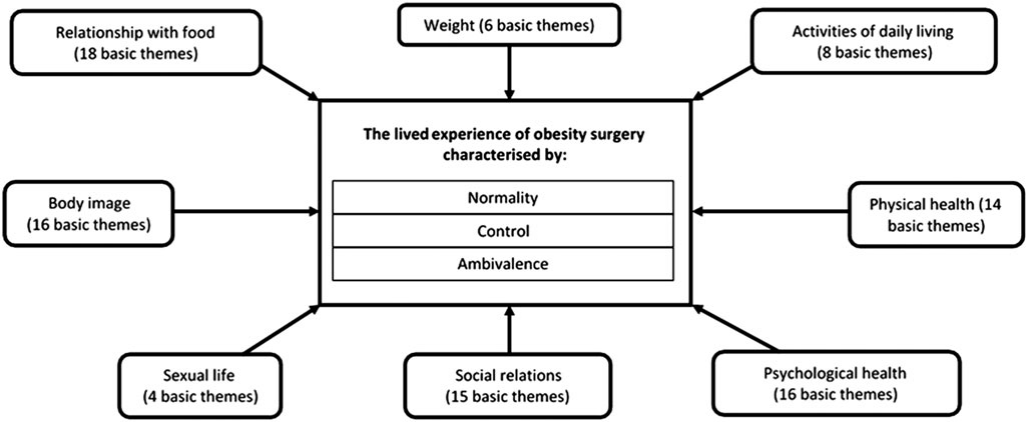
Thematic network describing the lived experience of obesity surgery.

Three global themes about the experience of living with the outcomes of bariatric surgery were identified (i) control; (ii) normality; and (iii) ambivalence. These reflected eight organizing themes encompassing a number of basic themes: ‘Weight’, ‘activities of daily living’, ‘physical health’, ‘psychological health’, ‘social relations’, ‘sexual life’, ‘body image’ and ‘eating behaviour and relationship with food’. The organizing themes and the basic themes they encompassed are presented in Table S1. A thematic network showing the organizing and global themes is provided in Fig. 3. Results are now presented under the three global themes with reference to the lower‐level organizing themes where appropriate. Direct participant quotes from the studies have been used to illustrate themes; when these have not been available, the words of the authors from the original studies are quoted to illustrate particular aspects.

### Control

Participants underwent bariatric surgery in the hopes of achieving better control of their eating, weight, health and lives. Central to this was being in control of eating and weight. The synthesis identified a global theme of control, which was reflected across all eight organizing themes. Initially after surgery, many participants reported not being able to tolerate much food and experiencing unpredictable gastrointestinal side effects or a temporary ‘loss of body control’ when eating 19, 22, 23, 24, 51, 69, 70, 71, 72, 74. Some participants also noticed that their taste for certain foods changed 23, 53. It was a challenge to learn how their body now tolerated food, and it took time to figure this out:

*‘You just have to find out how much you actually can eat and what you can tolerate….It has been some challenge navigating, such a labyrinth…’. [Female, Denmark]* 72

The majority of participants in the studies lost a large amount of weight rapidly in the first 6 to 12 months after surgery. This time period was likened to a ‘honeymoon period’^22^ (p.197), with participants reporting they felt ‘excited’^22^ (p.160) and ‘invincible’^53^ during this time. However, a few participants worried that the weight loss was too quick or ‘progressive’ and were concerned that they could not ‘influence it’ 70. Surgery was described as providing ‘structure’ 44, or a physical control over eating, also described as ‘stomach control’ 67. This included reduced hunger, improved satiety and physical side effects (such as dumping syndrome) if they ate too much. They appreciated this ‘external control’ 23, as prior to surgery they had been unable to control food themselves:

*‘Now I feel that the control is taken out of my hands. I didn't have that control over my body because my stomach controlled everything. If I eat too much I'm sick so I don't have the control anymore . . . that's a good thing because I couldn't control on my own’. [Female, UK]* 23

This seemed to allow participants to feel more in control of other areas of their lives, which contributed to their overall happiness:

*‘My self‐image is so different I project who I really am because I'm in control of my food and my exercise, control of my own schedule. […] I'm physically, emotionally and mentally in a better place’. [Participant, USA]* 24

However, many participants reported that after the first post‐operative year, the ‘stomach control’ imposed by the surgery started to wear off, and they were gradually able to eat more as time went on, although not as much as pre‐operatively. This meant they had to rely more on their own ‘head control’^67^ to manage their eating, and sticking to a healthy diet became increasingly difficult. For some, this led to weight re‐gain, or the fear of re‐gaining weight and subsequently feeling less confident that they could control other areas of their lives:

*‘I must admit that I'm quite scared, and often think, ‘What if my weight increases again?’ […] It's the worst case, like a nightmare. […] I've spoken to others who've told me that they've put on weight after two years. I get really anxious when they tell me this’. [Participant, Norway]* 67

Nine studies described patients who had experienced some weight re‐gain after the initial good weight loss 19, 20, 22, 26, 28, 60, 68, 69, 71. This led to feelings of ‘shame’ and ‘failure’ 26, 60.

Participants who re‐gained weight described relapsing into emotional eating or using food to cope with stress:

*‘[…] there was a lot of problems with my husband and my daughter who didn't get on and I was depressed over it […] and we had money problems and what have you and my way of coping was eating’. [Female, UK]* 20

Many studies reported that participants started to come to the realization that the surgery was a ‘life change, not just a crutch’^45^ and that they had to focus on eating healthier lifelong:

*‘Right after the surgery, there is this part of you that thinks, “I'm cured. I'm automatically going to lose weight.” But the surgery alone only works by itself for the first several months, maybe a year. But then you have to take over. You have to establish your new habits and your new patterns, and that can be rough because you are confronting a lot of issues that you never confronted before’. [Female, USA]* 22 *(p.207)*

### Normality

Throughout all aspects of their lives, normality was something that participants were striving for after having bariatric surgery. Participants described wanting lives that were less burdened by physical and psychological problems, a more normal or socially acceptable appearance, to be able to engage in normal everyday activities and have the same social and work opportunities and expectations for their lives that they felt others did. In some aspects of life, participants in the studies did indeed describe feeling more ‘normal’ after the surgery. For example, many reported experiencing less physical health problems and required less medications:

*‘I have arthritis and used to take four different pills. Now I don't have to take any pills. I used to have high blood pressure as well and took an additional two pills for that. I had a tray filled with pills’. [Female, Norway]* 66

A dramatic improvement in undertaking usual activities of daily living was reported for most participants in the studies. This included an improved ability to undertake domestic chores and carry out personal hygiene, and the ability to fit into seats in public settings:

*‘You had to walk into a restaurant and ask for a chair rather than a booth. My most exciting thing is just sitting in a booth’. [Participant, USA]* 24

The participants also reported improved work opportunities including an improved ability to carry out work tasks and better recognition and interactions with colleagues:

*‘Suddenly where I work, at meetings and suchlike: […] What do you think about this …….. So they listen to me more. People ask me questions. They consult me. And I don't need to shout loud any more because I can talk and they hear me anyway. So…it's an…almost measurable difference…because the expectations to overweight people are rather low…. a bit hurtful. That's how I felt’. [Female, Norway]* 54

Participants were pleased that they were now a more ‘average’ weight or looked more ‘normal’ 23, 65, 67, 73, 74. They appreciated the freedom to shop at a wider range of stores and increased clothing choices that they could use to draw attention to their new bodies:

*‘I wear the clothes I like, for example, red, because it draws attention, why not? We lose that fear of going into a shop and receiving comments’. [Female, Brazil]* 65

Some enjoyed the fact that they now blended in like ‘a normal person’ or were just ‘another face in the crowd’, as if they had never been obese ‘abnormal’ members of society 23, 24, 65, 69, 74.

However, in other aspects of their lives, participants' sense of normality was challenged. Many participants reported that initially their view of their body was not in sync with the reality of how much weight they had lost or with other people's views of their body:

*‘It's how you look at yourself, you still think that you're big, and even if you hear many comments like oh, you are looking so good and so on, and of course it helps a great deal, but the image of myself when looking in the mirror is that my belly is still big and so, ah, I still think it's hard’. [Participant, Sweden]* 60

Other changes after surgery that challenged their sense of normality included the development of unpleasant gastrointestinal symptoms (e.g. vomiting and diarrhoea), not being able to eat like others and the development of loose‐hanging excess skin:

*‘Eating in public often now attracts attention. […] Now people comment on how little I am eating, and the time it takes me to eat’. [Female, New Zealand]* 59

*‘[…] I have to kind of get myself put back to what I consider to be normal. Normal is not too skinny and it's certainly not fat, but it's not the hanging skin either’. [Male, Canada]* 73

Excess skin caused psychological problems for some who felt ‘depressed’ as a result of their ‘severe body hatred’ 27, 54, 56, 61, 65, 69, 73. Some felt that the excess skin was worse than being fat and sought plastic surgery to remove it as this was the only way they could finally look ‘normal’ 22, 24, 52, 54, 56, 61, 62, 65, 67, 69, 73, 74.

### Ambivalence

A global theme of ambivalence was identified around the lived experience of bariatric surgery, which was evident throughout all the studies and organizing themes. This is reflected in the participants' co‐existing accounts of how some things in their lives changed for the better, and other changes were difficult to cope with or adapt to. Many of the studies reported that patients experienced an improvement in several aspects of their physical health, which included improved mobility, reduction in pain, improvement in bariatric‐related comorbidities such as diabetes, a reduction in medications needed and improved fertility 22, 23, 24, 44, 45, 46, 51, 52, 55, 59, 60, 61, 62, 66, 67, 68, 69, 70, 71, 72, 74. Conversely, surgery also led to some negative changes to physical health such as the development of nutritional deficiencies and unpleasant gastrointestinal symptoms:

*‘…the women emphasized how their blood values and vitamin levels had changed dramatically after the surgery. […] they constantly struggled with iron deficiency, low haemoglobin percentage, and B12‐deficiency. While these levels were previously regarded as “normal” in terms of medical standards, they were far below the accepted level after the surgery’. [Authors' words]* 26

Although most studies reported that, overall, participants' were ‘far healthier’ following surgery and could ‘deal’ with the new problems 24, some felt that their physical health was worse:

*‘It feels like I have a rock in the machinery which makes me disabled in my daily life […] I am struggling with low blood pressure […] occasionally I see stars and nearly faint when I work’. [Female, Norway]* 26

Similarly, psychological changes were reported as both positive and negative. Participants often reported improved depression, confidence, self‐esteem and a sense of control over their life 67. In contrast, some participants found problems with low self‐esteem and confidence continued to ‘drag on’ and recognized that these long‐standing problems were not ‘going to be cured in a day’^22^ (p.151). The psychological need to eat remained for some despite being physically unable to eat as much as before:

*‘You're actually not hungry when you eat. Your brain keeps telling you that you are hungry. The stomach on the contrary is about to burst… This need, it's not just removed in surgery…There's such a psychological need all the time’. [Female, Denmark]* 72

This meant they had to learn to deal differently with difficult emotions that previously they had used food to cope with:

*‘I now have to deal with problems that I always fed with my addiction to food. Sometimes I don't really want to have to deal with these problems. It was much easier to just shove them to the side, pat them down, and cover them with food and move onto something else’. [Female, USA]* 22 *(p.162)*

Some participants said that losing weight forced them to re‐discover who they were as a person, which could be a difficult process:

*‘You have to be psychologically ready for this surgery because it forces you to look inside yourself, and that can be very hard’. [Female, USA]* 22 *(p.177)*

For some, the weight had served as some ‘protection’ against the outside world^22^ (p.170), and they felt vulnerable and defenceless as they lost weight 27, 54, 56, 69, 74.

### Social issues

After losing weight with surgery, many participants commented that they received more positive social feedback; people no longer ‘avoided’ them, and they felt more confident to engage in social activities^22^, ^24^, ^66^, ^73^:

*‘I use to go to a party and find a chair and that's where I would stay the entire party. Now I go to a party and I go around visit with everybody and have a good old time. I was too embarrassed to even move around because I didn't want anyone to notice me’. [Participant, USA]* 24

However, some felt resentment or ‘conflict’ about this improved attention as it made them realize just how badly they had been treated when they were obese:

*‘You flip between thinking, “Well, I wasn't good enough for you before, so I don't want you around now,” to wanting to embrace the supposedly new you and the new reactions to you’. [Female, USA]* 22 *(p.87)*

Some participants also received negative attention from others who thought they had taken the ‘easy way out’ by having surgery:

*‘As the daughter of one of the participants commented, “It's not really an achievement the same as if it had been done normally. You've just had your insides cut up and it doesn't let you eat. Anyone could do it [lose weight].” ’* 49

The impact of the surgery on participants' romantic and sexual lives also demonstrated the ambivalence with which many participants regarded their experience of bariatric surgery. Some participants reported more romantic or sexual attention and had more opportunities for romantic relationships:

*‘Men are starting to look at me differently which is kind of fun and I'm going out with a nice guy from school who really treats me like a lady’. [Female, USA]* 52

Some found the new romantic attention ‘scary’ and described how they did not have the experience or knowledge to deal with this kind of attention 22, 24, 46, 52. These new social possibilities required the development of new social skills that they had not previously had to use:

*‘I was 225 pounds in the eighth grade when girls are experimenting with boys. I was never involved in conversations about boys because I was not having those experiences. So basically, as an adult after the weight loss, when relationships were a possibility, I thought about them the way a thirteen year old would. Physically and professionally, I was in my thirties, but emotionally I was a teenager when it came to relational issues’. [Female, USA]* 22 *(p.75)*

## Discussion

Bariatric surgery is the most clinically effective treatment for severe and complex obesity, in terms of both weight loss and the improvement of weight‐related comorbidities. However, it leads to impacts on several other areas of patients' lives, which are important to consider. This systematic review of qualitative research studies synthesized what is currently known about the patient perspective of living with the outcomes of bariatric surgery. The synthesis demonstrated that bariatric surgery led to a number of changes to the lives of participants, including their weight, activities of daily living, physical health, psychological health, social relations, sexual life, body image and eating behaviour and relationship with food. Three global themes (control, normality and ambivalence) were identified, which describe the lived experience of bariatric surgery throughout all these areas of participants' lives. Participants were striving for control of their food, weight and health, as well as developing a new identity as a ‘normal’ or socially acceptable person. Although many of the changes after surgery were reported as being positive and led to participants feeling more normal and in control of their lives, some problems were also experienced. Other changes were seen as neither positive nor negative but were challenging and required adaptation, which contributed to the overall ambivalent nature of many accounts of life after bariatric surgery. These data are important because patients deciding to undergo surgery need to be aware of both the positive and challenging nature of changes reported and need to be supported appropriately in the long term.

Many of the findings from this qualitative synthesis relate to the psychosocial issues faced by patients after undergoing bariatric surgery and have not been identified in the quantitative literature describing outcomes of bariatric surgery. Thus, this synthesis provides important new insights around patients' experiences of bariatric surgery as a whole. In particular, it demonstrates that the effect of bariatric surgery on psychosocial outcomes is far from straightforward. Living with the changes caused by bariatric surgery is complicated, unstable and requires ongoing negotiation. Participants in the included studies initially felt more in control of weight and eating, but this sense of control diminished as time progressed. It is well known that some patients will re‐gain a certain amount of weight between 1 and 10 years post‐surgery 15. The findings of this qualitative synthesis highlight that weight re‐gain can be associated with a feeling of loss of control and a negative psychological experience for patients.

The findings from our synthesis help to provide explanatory background to previous studies evaluating the impact of bariatric surgery on HRQL, which is often reported poorly 12, 75. Where impact on HRQL has been reported, there is significant variation in results, with improvements in some (e.g. physical functioning) but not all (e.g. social and emotional functioning) areas 12, 76, 77, 78. This synthesis has highlighted that although patients in the included studies reported some positive psychological changes such as reduced depression and improved self‐confidence, they also experienced difficulties creating a new identity and developing new coping strategies that did not involve food. Meana and Ricciardi termed these ‘tension‐generating changes’; changes that were neither clearly positive nor negative but required a process of adaptation 21, 22 (p.209). A recent study by Wood and Ogden interviewed people who underwent bariatric surgery more than 8 years ago 79. Those who had maintained good weight loss had been more able to ‘functionalize’ food (e.g. ‘eat to live’ rather than ‘live to eat’ 23, 24) and develop new coping strategies and a more positive self‐image 79.

This synthesis also highlighted that patients who underwent bariatric surgery alternated between feeling they were a more ‘normal’, socially acceptable person and less ‘normal’ because of the development of loose‐hanging excess skin, which impacted on their body image and relationships with others. Some participants said they desired plastic surgery as a way to finally achieve normality. Ogden *et al.* recently published a study on patients' experiences of plastic surgery to remove excess skin following bariatric surgery 80. They reported that after undergoing both bariatric and plastic surgery, participants' came to the realization that their psychological issues remained untreated and that their physical shape may not have been the key factor contributing to their negative self‐image 80. In this review, it was shown that people who had undergone bariatric surgery may also be presented with a new range of social opportunities, some of which can be frightening (e.g. romantic prospects) as they may be outside the scope of their previous experience. These findings highlight the importance of concurrent psychological support alongside surgery.

### Clinical implications and future research

This synthesis emphasizes the need for health professionals to help patients manage the changes and challenges resulting from bariatric surgery. Patients need ongoing support in relation to their sense of control and normality and to help them navigate tensions inherent within the outcomes experienced. The widespread and complex nature of the changes experienced by the participants in the included studies, even several years after bariatric surgery, reinforces the view that obesity is a chronic disease that can be treated but never totally ‘cured’ 81. Therefore, patients require access to life‐long support to manage their obesity and maximize the benefit of their treatments. In particular, it is important for bariatric surgery services to ensure access to long‐term dietary and psychological support to help patients navigate the challenging aspects of life post‐surgery, for example, coping with a return of hunger feelings and control of eating, weight re‐gain and changes to personal identity and body image. Health professionals need to help patients to recognize when a small amount of weight re‐gain is normal, vs. when it is more problematic, and provide additional support in these instances.

Most health professionals working with patients who have undergone bariatric surgery recognize that good follow‐up care is essential in achieving a successful outcome 82, 83, 84. Despite this, follow‐up care after bariatric surgery varies greatly 85, 86, 87. Guidance relating to the long‐term follow‐up care of bariatric surgery patients mainly focuses on the monitoring and treatment of physical symptoms, comorbidities and nutritional deficiencies, with little guidance on how to help patients cope with weight re‐gain, continued control of eating and other psychological issues following surgery 13, 84, 88. There is a need for further research to develop and assess interventions to support patients with long‐term weight maintenance and to cope with the profound changes to their lives after this surgery, particularly to their eating habits and psychological health. The findings of this synthesis are useful for health professionals and policy makers working in bariatric surgery services to inform the future development of these services to be more in line with patient needs. Improved follow‐up care services may lead to better outcomes of bariatric surgery, which would mean cost savings to health care in managing obesity‐related health problems.

### Strengths and limitations of this synthesis

This qualitative synthesis is, to the authors' knowledge, the first attempt to synthesize the wealth of qualitative literature that exists in the field of bariatric surgery. There is an increasing recognition that qualitative research studies need to be synthesized to inform policy and practice as is often performed for quantitative studies 40. As a large number of studies conducted in different countries were identified and synthesized, this synthesis will help future qualitative researchers focus their research questions on areas less well explored and will also help to provide recommendations for future clinical practice. A limitation of this review is that a single reviewer initially screened records, rather than two reviewers, which could have led to the possible exclusion of relevant articles. However, any queries on inclusion or exclusion were discussed with a second reviewer who also cross‐checked that all full‐text articles selected for inclusion met the inclusion criteria. The rigour of the synthesis was enhanced by a second reviewer completing independent dual data extraction on a sample of included studies. Preliminary thematic networks were also discussed between three of the authors to discuss and agree the emergence of themes. A limitation of this synthesis is that we included only published data and did not include unpublished data or ‘grey literature’.

## Conclusion

This systematic review and thematic synthesis has highlighted the profound long‐term impact that bariatric surgery has on many different aspects of people's lives and the challenges they experience in coming to terms with these changes. The overarching themes of control, normality and ambivalence characterized the complexity of changes experienced. Participants came to the realization that to achieve and maintain positive outcomes from the surgery, they had to sustain life‐long changes to their eating habits, which were much more difficult than anticipated. Surgery was not the end of their journey with obesity but rather the beginning of a new and sometimes challenging path. These findings contribute to the knowledge base about the long‐term outcomes of obesity surgery and demonstrate the importance of ongoing support to help people negotiate these challenges and maintain the positive changes achieved.

## Conflict of interest statement

The authors have no conflicts of interest to declare.

## Supporting information

Supporting Information: Search strategies for systematic review and synthesis of qualitative studies.
Table S1: Organising themes and their associated basic themes in thematic synthesis.
