# Supplementary material for: Patient experiences of outcomes of bariatric surgery: a systematic review and qualitative synthesis
Source: Obes Rev. 2017 Mar 8;18(5):547–59. doi: 10.1111/obr.12518 (PMC5709707; doi:10.1111/obr.12518)
Supplement: Supplementary file 1 — Supporting Information: Search strategies for systematic review and synthesis of qualitative studies. Table S1: Organising themes and their associated basic themes in thematic synthesis. [file OBR-18-547-s001.zip › SI search strategies.docx]

Appendix 1: Search strategies for systematic review and synthesis of qualitative studies

**Medline and Embase (using Ovid)**

| 1. | exp Obesity/ |
| --- | --- |
| 2. | Overweight/ |
| 3. | over weight.tw. |
| 4. | overweight.tw. |
| 5. | over eating.tw. |
| 6. | overeating.tw. |
| 7. | Weight Loss/ |
| 8. | weight loss.tw. |
| 9. | weight reduc$.tw. |
| 10. | (obese or obesity).tw. |
| 11. | or/1-10 |
| 12. | exp Bariatric Surgery/ |
| 13. | bariatric.tw. |
| 14. | (antiobesity adj3 surg$).tw. |
| 15. | (obesity adj3 surg$).tw. |
| 16. | "restrictive surgery".tw. |
| 17. | gastroplasty.tw. |
| 18. | (gastrogastrostomy or gastro gastrostomy).tw. |
| 19. | jejuno-ileal bypass.tw. |
| 20. | jejunoileal bypass.tw. |
| 21. | gastrointestinal diversion$.tw. |
| 22. | Biliopancreatic Diversion/ |
| 23. | biliopancreatic diversion.tw. |
| 24. | bilio-pancreatic diversion.tw. |
| 25. | ((biliopancreatic or bilio-pancreatic) adj1 bypass).tw. |
| 26. | gastric band$.tw. |
| 27. | silicon band$.tw. |
| 28. | exp Gastroenterostomy/ |
| 29. | sleeve gastrectomy.tw. |
| 30. | gastric sleeve.tw. |
| 31. | LAGB.tw. |
| 32. | stomach stapl$.tw. |
| 33. | lap band$.tw. |
| 34. | lapband$.tw. |
| 35. | malabsorptive surg$.tw. |
| 36. | mason$ procedure.tw. |
| 37. | Anastomosis, Roux-en-Y/ |
| 38. | Roux-en-Y.tw. |
| 39. | malabsorptive procedure$.tw. |
| 40. | duodenal switch$.tw. |
| 41. | or/12-40 |
| 42. | 11 and 41 |
| 43. | exp Obesity/su [Surgery] |
| 44. | 42 or 43 |
| 45. | Focus Groups/ |
| 46. | focus group$1.tw. |
| 47. | Interview/ |
| 48. | interview$.tw. |
| 49. | exp Interviews as Topic/ |
| 50. | ethnograph$.tw. |
| 51. | content analysis.tw. |
| 52. | grounded theory.tw. |
| 53. | grounded approach.tw. |
| 54. | qualitative.tw. |
| 55. | Qualitative Research/ |
| 56. | phenomenolog$.tw. |
| 57. | discourse analysis.tw. |
| 58. | constant comparison.tw. |
| 59. | constant comparative method.tw. |
| 60. | observational method$.tw. |
| 61. | theoretical sampl$.tw. |
| 62. | thematic analys?s.tw. |
| 63. | observation$.tw. |
| 64. | exp Observation/ |
| 65. | or/45-64 |
| 66. | 44 and 65 |

**Medline In-Process (using Ovid)**

| 1. | over weight.tw. |
| --- | --- |
| 2. | overweight.tw. |
| 3. | over eating.tw. |
| 4. | overeating.tw. |
| 5. | weight loss.tw. |
| 6. | weight reduc$.tw. |
| 7. | (obese or obesity).tw. |
| 8. | or/1-7 |
| 9. | bariatric.tw. |
| 10. | (obesity adj3 surg$).tw. |
| 11. | "restrictive surgery".tw. |
| 12. | gastroplasty.tw. |
| 13. | (gastrogastrostomy or gastro gastrostomy).tw. |
| 14. | jejuno-ileal bypass.tw. |
| 15. | jejunoileal bypass.tw. |
| 16. | biliopancreatic diversion.tw. |
| 17. | bilio-pancreatic diversion.tw. |
| 18. | ((biliopancreatic or bilio-pancreatic) adj1 bypass).tw. |
| 19. | gastric band$.tw. |
| 20. | silicon band$.tw. |
| 21. | sleeve gastrectomy.tw. |
| 22. | gastric sleeve.tw. |
| 23. | LAGB.tw. |
| 24. | lap band$.tw. |
| 25. | Roux-en-Y.tw. |
| 26. | malabsorptive procedure$.tw. |
| 27. | duodenal switch$.tw. |
| 28. | or/9-27 |
| 29. | 8 and 28 |
| 30. | focus group$1.tw. |
| 31. | Interview/ |
| 32. | interview$.tw. |
| 33. | ethnograph$.tw. |
| 34. | content analysis.tw. |
| 35. | grounded theory.tw. |
| 36. | grounded approach.tw. |
| 37. | qualitative.tw. |
| 38. | phenomenolog$.tw. |
| 39. | discourse analysis.tw. |
| 40. | constant comparison.tw. |
| 41. | constant comparative method.tw. |
| 42. | observational method$.tw. |
| 43. | theoretical sampl$.tw. |
| 44. | thematic analys?s.tw. |
| 45. | observation$.tw. |
| 46. | or/30-45 |
| 47. | 29 and 46 |

**PsycINFO (using Ovid)**

| 1. | exp Obesity/ |
| --- | --- |
| 2. | Overweight/ |
| 3. | over weight.tw. |
| 4. | overweight.tw. |
| 5. | over eating.tw. |
| 6. | overeating.tw. |
| 7. | Weight Loss/ |
| 8. | weight loss.tw. |
| 9. | weight reduc$.tw. |
| 10. | (obese or obesity).tw. |
| 11. | or/1-10 |
| 12. | exp Bariatric Surgery/ |
| 13. | bariatric.tw. |
| 14. | (obesity adj3 surg$).tw. |
| 15. | "restrictive surgery".tw. |
| 16. | gastroplasty.tw. |
| 17. | jejuno-ileal bypass.tw. |
| 18. | jejunoileal bypass.tw. |
| 19. | biliopancreatic diversion.tw. |
| 20. | gastric band$.tw. |
| 21. | sleeve gastrectomy.tw. |
| 22. | gastric sleeve.tw. |
| 23. | LAGB.tw. |
| 24. | lap band$.tw. |
| 25. | malabsorptive surg$.tw. |
| 26. | Roux-en-Y.tw. |
| 27. | malabsorptive procedure$.tw. |
| 28. | duodenal switch$.tw. |
| 29. | or/12-28 |
| 30. | 11 and 29 |
| 31. | exp "obesity (attitudes toward)"/ |
| 32. | exp client attitudes/ |
| 33. | exp group discussion/ |
| 34. | focus group$1.tw. |
| 35. | exp interviewing/ |
| 36. | interview$.tw. |
| 37. | exp ethnography/ |
| 38. | ethnograph$.tw. |
| 39. | exp Content Analysis/ |
| 40. | content analysis.tw. |
| 41. | exp Grounded Theory/ |
| 42. | grounded theory.tw. |
| 43. | grounded approach.tw. |
| 44. | qualitative.tw. |
| 45. | exp Qualitative Research/ |
| 46. | exp phenomenology/ |
| 47. | phenomenolog$.tw. |
| 48. | exp discourse analysis/ |
| 49. | discourse analysis.tw. |
| 50. | constant comparison.tw. |
| 51. | constant comparative method.tw. |
| 52. | exp Observation Methods/ |
| 53. | observational method$.tw. |
| 54. | theoretical sampl$.tw. |
| 55. | thematic analys?s.tw. |
| 56. | observation$.tw. |
| 57. | or/31-56 |
| 58. | 30 and 57 |

**Cochrane library**

| 1. | MeSH descriptor: [Obesity] explode all trees |
| --- | --- |
| 2. | MeSH descriptor: [Overweight] this term only |
| 3. | over weight:ti,ab |
| 4. | overweight:ti,ab |
| 5. | over eating:ti,ab |
| 6. | overeating:ti,ab |
| 7. | MeSH descriptor: [Weight Loss] this term only |
| 8. | weight loss:ti,ab |
| 9. | weight reduc*:ti,ab |
| 10. | (obese or obesity):ti,ab |
| 11. | (#1 or #2 or #3 or #4 or #5 or #6 or #7 or #8 or #9 or #10) |
| 12. | MeSH descriptor: [Bariatric Surgery] explode all trees |
| 13. | bariatric:ti,ab |
| 14. | (obesity near/3 surg*):ti,ab |
| 15. | "restrictive surgery":ti,ab |
| 16. | gastroplasty:ti,ab |
| 17. | (gastrogastrostomy or gastro gastrostomy):ti,ab |
| 18. | jejuno-ileal bypass:ti,ab |
| 19. | jejunoileal bypass:ti,ab |
| 20. | gastrointestinal diversion*:ti,ab |
| 21. | MeSH descriptor: [Biliopancreatic Diversion] this term only |
| 22. | biliopancreatic diversion:ti,ab |
| 23. | ((biliopancreatic or bilio-pancreatic) near/1 bypass):ti,ab |
| 24. | gastric band*:ti,ab |
| 25. | silicon band*:ti,ab |
| 26. | MeSH descriptor: [Gastroenterostomy] explode all trees |
| 27. | sleeve gastrectomy:ti,ab |
| 28. | gastric sleeve:ti,ab |
| 29. | LAGB:ti,ab |
| 30. | stomach stapl*:ti,ab |
| 31. | lap band*:ti,ab |
| 32. | lapband:ti,ab |
| 33. | malabsorptive surg*:ti,ab |
| 34. | mason* procedure:ti,ab |
| 35. | MeSH descriptor: [Anastomosis, Roux-en-Y] this term only |
| 36. | Roux-en-Y:ti,ab |
| 37. | malabsorptive procedure*:ti,ab |
| 38. | duodenal switch*:ab,ti |
| 39. | (#12 or #13 or #14 or #15 or #16 or #17 or #18 or #19 or #20 or #21 or #22 or #23 or #24 or #25 or #26 or #27 or #28 or #29 or #30 or #31 or #32 or #33 or #34 or #35 or #36 or #37 or #38) |
| 40. | (#11 and #39) |
| 41. | MeSH descriptor: [Obesity] explode all trees and with qualifier(s): [Surgery - SU] |
| 42. | (#40 or #41) |
| 43. | Focus Groups/ |
| 44. | focus group*:ti,ab |
| 45. | Interview/ |
| 46. | interview*:ti,ab |
| 47. | exp Interviews as Topic/ |
| 48. | ethnograph*:ti,ab |
| 49. | content analysis:ti,ab |
| 50. | grounded theory:ti,ab |
| 51. | grounded approach:ti,ab |
| 52. | qualitative:ti,ab |
| 53. | Qualitative Research/ |
| 54. | phenomenolog*:ti,ab |
| 55. | discourse analysis:ti,ab |
| 56. | constant comparison:ti,ab |
| 57. | constant comparative method:ti,ab |
| 58. | observational method*:ti,ab |
| 59. | theoretical sampl*:ti,ab |
| 60. | thematic analys*s:ti,ab |
| 61. | observation*:ti,ab |
| 62. | exp Observation/ |
| 63. | 43 or 44 or 45 or 46 or 47 or 48 or 49 or 50 or 51 or 52 or 53 or 54 or 55 or 56 or 57 or 58 or 59 or 60 or 61 or 62 |
| 64. | #42 and #63 |

**CINAHL (using EBSCOhost)**

| 1. | (MH "Obesity+") |
| --- | --- |
| 2. | TI over weight or AB over weight |
| 3. | TI overweight or AB overweight |
| 4. | TI over eating or AB over eating |
| 5. | TI overeating or AB overeating |
| 6. | (MH "Weight Loss") |
| 7. | TI weight loss or AB weight loss |
| 8. | TI weight reduc* or AB weight reduc* |
| 9. | TI ( (obese or obesity) ) or AB ( (obese or obesity) ) |
| 10. | S1 or S2 or S3 or S4 or S5 or S6 or S7 or S8 or S9 |
| 11. | (MH "Bariatric Surgery+") |
| 12. | TI bariatric or AB bariatric |
| 13. | TI (antiobesity N3 surg*) or AB (antiobesity N3 surg*) |
| 14. | TI (obesity N3 surg*) or AB (obesity N3 surg*) |
| 15. | TI "restrictive surgery" or AB "restrictive surgery" |
| 16. | TI gastroplasty or AB gastroplasty |
| 17. | TI jejuno-ileal bypass or AB jejuno-ileal bypass |
| 18. | TI jejunoileal bypass or AB jejunoileal bypass |
| 19. | TI biliopancreatic diversion* or AB biliopancreatic diversion* |
| 20. | TI bilio-pancreatic diversion* or AB bilio-pancreatic diversion* |
| 21. | TI gastric band* or AB gastric band* |
| 22. | (MH "Gastroenterostomy+") |
| 23. | TI sleeve gastrectomy or AB sleeve gastrectomy |
| 24. | TI LAGB or AB LAGB |
| 25. | TI stomach stapl* or AB stomach stapl* |
| 26. | TI lap band* or AB lap band* |
| 27. | TI malabsorptive surg* or AB malabsorptive surg* |
| 28. | (MH "Anastomosis, Roux-en-Y") |
| 29. | TI Roux-en-Y or AB Roux-en-Y |
| 30. | TI malabsorptive procedure* or AB malabsorptive procedure* |
| 31. | TI duodenal switch* or AB duodenal switch* |
| 32. | (S11 or S12 or S13 or S14 or S15 or S16 or S17 or S18 or S19 or S20 or S21 or S22 or S23 or S24 or S25 or S26 or S27 or S28 or S29 or S30 or S31) |
| 33. | S10 and S32 |
| 34. | (MH "Obesity+/SU") |
| 35. | S33 or S34 |
| 36. | (MH "Focus Groups") |
| 37. | TX focus group |
| 38. | (MH "Interviews+") |
| 39. | TX interview |
| 40. | TX ethnograph* |
| 41. | (MH "Ethnographic Research") |
| 42. | TX content analysis |
| 43. | (MH "Content Analysis") |
| 44. | TX grounded theory |
| 45. | (MH "Grounded Theory") |
| 46. | TX grounded approach |
| 47. | TX qualitative |
| 48. | (MH "Qualitative Studies+") |
| 49. | TX phenomenolog* |
| 50. | (MH "Phenomenology") |
| 51. | (MH "Phenomenological Research") |
| 52. | TX discourse analysis |
| 53. | (MH "Discourse Analysis") |
| 54. | TX constant comparative method |
| 55. | (MH "Constant Comparative Method") |
| 56. | TX observational method |
| 57. | (MH "Observational Methods+") |
| 58. | TX theoretical sampl* |
| 59. | (MH "Theoretical Sample") |
| 60. | (MH "Purposive Sample") |
| 61. | TX thematic analys?s |
| 62. | (MH "Thematic Analysis") |
| 63. | TX observation* |
| 64. | TX constant comparison |
| 65. | S36 or S37 or S38 or S39 or S40 or S41 or S42 or S43 or S44 or S45 or S46 or S47 or S48 or S49 or S50 or S51 or S52 or S53 or S54 or S55 or S56 or S57 or S58 or S59 or S60 or S61 or S62 or S63 or S64 |
| 66. | S35 and S65 |

**Web of Science (including Science Citation Index Expanded, Social Sciences Citation Index, and Arts & Humanities Citation Index)**

| 1. | TS = ((obese OR obesity) OR overweight OR "over weight" OR overeating OR over eating OR "weight loss" OR "weight reduc*") |
| --- | --- |
| 2. | TS = ("duodenal switch*" OR "malabsorptive procedure*" OR "Roux-en-Y" OR "mason* procedure" OR "malabsorptive surg*" OR "lapband*" OR "lap band*" OR "stomach stapl*" OR LAGB OR "gastric sleeve" OR "sleeve gastrectomy" OR gastroenterostomy OR "silicon band" OR "gastric band*" OR "bilio-pancreatic bypass" OR "biliopancreatic bypass" OR "bilio-pancreatic diversion" OR "biliopancreatic diversion" OR "gastrointestinal diversion*" OR "jejunoileal bypass" OR "jejuno-ileal bypass" OR (gastrogastrostomy OR "gastro gastrostomy") OR gastroplasty OR "restrictive surgery" OR (obesity SAME surg*) OR (antiobesity SAME surg*) OR bariatric OR "bariatric surg*") |
| 3. | #2 AND #1 |
| 4. | TS=("focus group$" OR interview* OR ethnograph* OR "content analysis" OR "grounded theory" OR "grounded approach" OR qualitative OR "qualitative research" OR phenomenolog* OR "discourse analysis" OR "constant comparison" OR "constant comparative method" OR "observational method*" OR "theoretical sampl*" OR "thematic analys$s" or observation*) |
| 5. | #4 AND #3 |
