# Supplementary material for: Patient experiences of outcomes of bariatric surgery: a systematic review and qualitative synthesis
Source: Obes Rev. 2017 Mar 8;18(5):547–59. doi: 10.1111/obr.12518 (PMC5709707; doi:10.1111/obr.12518)
Supplement: Supplementary file 1 — Supporting Information: Search strategies for systematic review and synthesis of qualitative studies. Table S1: Organising themes and their associated basic themes in thematic synthesis. [file OBR-18-547-s001.zip › Table S1 all basic themes.docx]

Appendix X: Organising themes and their associated basic themes in qualitative synthesis

| **Weight**  **(6 basic themes)** | **Activities of daily living**  **(8 basic themes)** | **Physical health (14 basic themes)** | **Psychological health**  **(16 basic themes)** | **Social relations**  **(15 basic themes)** | **Sexual life**  **(4 basic themes)** | **Body image**  **(16 basic themes)** | **Eating behaviour and relationship with food**  **(18 basic themes)** |
| --- | --- | --- | --- | --- | --- | --- | --- |
| Pleased with initial weight loss | Ability to fit into public spaces more easily | Improvement of co-morbidities/health | Improved mental health, reduction in depression | No longer rejected/discriminated against by society | Improved sex life | More freedom with clothing choices | Food intolerances and side-effects associated with eating, loss of body control in early stages |
| Weight loss too quick | More able to carry out household chores/tasks | Improved fertility | Improved self-confidence, self-esteem | Less visible as 'bodies', more visible as individuals | Mixed changes on sex life | Pleased to get rid of old clothes | Lack of hunger or cravings in early stages |
| Felt had lost too much | More able to carry out personal hygiene or self-care | Less medications | Improved outlook on life | Less negative attention, now 'one of the crowd' | Negative impact on sex life due to loose skin | External transformation quicker than inner, 'mind-body lag' | Feelings of food deprivation in early stages |
| Wanted to lose more | Less planning required to undertake activities | Reduced pain | Personal satisfaction as woman | More positive attention from others, which caused emotional conflict for some | Sexual problems led to breakdown of relationships, psychological problems and reduced quality of life. | Engaged in more body checking behaviours in order to check if lost weight | Difficulties adjusting to new diet |
| Weight re-gain in the long-term | Improved sleep | More energy | A greater sense of control over life (internal control) | More sexual/romantic attention and opportunities, which was frightening for some | N/A | It was not until their cognitions changed and they perceived they were no longer obese that their emotions and behaviours started to change | Food required more effort and planning |
| Maintained weight but feared re-gain | Had to re-learn how to use hands, mouth, cutlery when eating | Improved mobility | Coping/adapting to new reality was challenging | Loose skin caused problems for romantic relationships | N/A | Please with improved appearance, and that looked 'normal' | After adaptation phase, surgery imposed 'external' or 'stomach' control over eating, provided more structure |
| N/A | Way they moved not in tune with smaller body | Improved fitness | Re-discovery of self/identity | Negative comments from others that had lost too much weight | N/A | Feeling that lost too much weight associated with negative body image | New feeling of satiety, separation of sensations between stomach and mind |
| N/A | Excess skin interfered with functioning, ADLs | Increased willingness and ability to take up exercise/sport | Loss of body control in initial stages caused feelings of helplessness and worry | Negative reactions from others that 'took the easy way out'/stigma of having surgery | N/A | Excess skin challenged their body image, which led to psychological problems, and the feeling that still weren't 'normal' | External control led to better internal control of eating |
| N/A | N/A | New medical problems since surgery, but still healthier | Difficulty maintaining control over food and weight in long-term leading to anxiety and fear | Improved socialisation/ social opportunities | N/A | Fear/avoidance of showing their body in public | Some did not get side-effects such as dumping syndrome, making control of food more challenging (less external control) |
| N/A | N/A | Severe complications and illness following surgery | Development of new addictive behaviours such as substance abuse | Withdrew from social activities due to side-effects/loose skin and fear of others reactions | N/A | Excess skin more problematic than former fatness | Change in relationship with food from 'living to eat' to 'eating to live' |
| N/A | N/A | Weakness initially, unable to do physical activity | Mixed emotions post-surgery | Initially withdrew from social activities, then engaged in more social activities and relationships once their head caught up with their body that they were indeed slimmer | N/A | Desire for plastic surgery to remove excess skin | Desire for food still present - satiety reached but satiation not |
| N/A | N/A | Physical problems due to excess skin | Buyer's remorse' in early stages | Required to develop new social skills | N/A | Drastic measures taken in an attempt to qualify for funded plastic surgery | Loss of food as a coping mechanism, leading to disordered eating for some |
| N/A | N/A | Gastro-intestinal side-effects | Shame remained - shame of being fat replaced with shame of having had surgery | Positive and negative changes in family dynamics and close relationships | N/A | Avoidance of negative body image triggers, related to excess skin and mind-body lag | Still struggling with food as a coping strategy although easier than before surgery |
| N/A | N/A | Nutritional deficiencies | Felt vulnerable and defenceless as they lost weight which had been their protection against the world | Improvement in work life | N/A | Reframed negative loose skin by focusing on positive changes gained | Control harder to maintain as time went on - reduced effect of surgery |
| N/A | N/A | N/A | Disconnect between physical body and mind | Quit or lost job | N/A | Body image influenced by feedback from others - negative or positive | Difficulty adhering to post-op diet in the long-term |
| N/A | N/A | N/A | Still left with unresolved psychological problems | N/A | N/A | Social support critical in helping deal with body image changes | Portion sizes increased over time |
| N/A | N/A | N/A | N/A | N/A | N/A | N/A | Grazing and returning to old eating habits in long-term |
|  |  |  |  |  |  |  | Realisation that have to make and sustain dietary and lifestyle changes |
